# Mitochondria-targeting theranostics

**DOI:** 10.1186/s40824-018-0145-7

**Published:** 2018-11-08

**Authors:** Han Chang Kang

**Affiliations:** Department of Pharmacy, College of Pharmacy, The Catholic University of Korea, 43 Jibong-ro, Wonmi-gu, Bucheon-si, Gyeonggi-do 14662 Republic of Korea

**Keywords:** Diagnostics, Drug delivery, Mitochondria-targeting, Subcellular targeting, Theranostics

## Abstract

**Background:**

Interest in subcellular organelle-targeting theranostics is substantially increasing due to the significance of subcellular organelle-targeting drug delivery for maximizing therapeutic effects and minimizing side effects, as well as the significance of theranostics for delivering therapeutics at the correct locations and doses for diseases throughout diagnosis. Among organelles, mitochondria have received substantial attention due to their significant controlling functions in cells.

**Main body:**

With the necessity of subcellular organelle-targeting drug delivery and theranostics, examples of mitochondria-targeting moieties and types of mitochondria-targeting theranostics were introduced. In addition, the current studies of mitochondria-targeting theranostic chemicals, chemical conjugates, and nanosystems were summarized.

**Conclusion:**

With the current issues of mitochondria-targeting theranostic chemicals, chemical conjugates, and nanosystems, their potentials and alternatives are discussed.

## Background

The development of drug delivery systems (DDSs) has been initiated to solve the poor aqueous solubility of chemical drugs and the poor blood stability of protein and gene drugs. For this purpose, various micron-sized or nanosized DDSs, such as microspheres, micelles, and liposomes, have been investigated, and few clinically available products, such as Lupron Depot®, Genexol PM®, and Doxil®, respectively, are currently on the market [1]. However, their low functionalized characteristics have limited to improve the therapeutic effects and reduce the side effects of delivered drugs.

Thus, with certain terms, the roles of these DDSs have been expanded. First, the term “Targeting” has been introduced to maximize therapeutic effects and minimize unwanted effects of drugs because the places that require drugs (i.e., target sites) should be distinguished from other places (i.e., nontarget sites). Most targeted DDSs have been designed to selectively reach to the organs, tissues, and cells of interest by physiological, pathological, and anatomical differences and specific interactions between ligands on the targeted DDS and their counter receptors on the plasma membrane. Very recently, for deeper targeting than cellular levels, subcellular organelles have been attracted because the organelles are actual sites of the mode of action of drugs [2, 3]. Second, the terms “Imaging” and “Diagnosis” of imaging or diagnostic molecules have been introduced instead of “Therapy” of drugs because the sites and the severity of diseases should be known for the effective therapeutic effects of prescribed drugs and the payloads in delivery carriers are not only drugs but also imaging or diagnostic molecules. In particular, beyond “Imaging”, “Theranostics” (i.e., combined systems of “Therapy” and “Imaging/Diagnosis”) has been interested in future DDSs. Thus, theranostic DDSs at subcellular organelles could be the future in the field of DDSs. Among various subcellular organelle-targeting theranostics, this review focuses on mitochondria-targeting theranostic DDSs.

### Theranostics

Theranostics may be defined as a combined system of diagnostics and therapeutics in a formulation. Its significance is to reduce the “trial-and-error” process for identifying a correct medicine and then maximize the therapeutic effects of the medicine because diagnostics in theranostics can diagnose the locations and status of diseases in organs, tissues, or cells; moreover, therapeutics in theranostics can effectively treat the diseases. Thus, interest in theranostics with the dual functions of therapeutic efficacy and diagnosis/imaging is rapidly increasing.

The simultaneous delivery of both diagnostics and therapeutics can be performed using chemical conjugates or nanosized/micron-sized carriers. For chemical conjugates, diagnostics and therapeutics can be chemically linked to each other, at ends of certain linker molecules, or to water-soluble macromolecules. Moreover, in various nanosized or micron-sized carriers, diagnostics and therapeutics can be physically or chemically loaded. Currently, the wide variety of imaging molecules as diagnostics includes radioactive nuclides, optical probes, or metal chelates, which are detectable by positron emission tomography (PET)/single photon emission computed tomography (SPECT), fluorescence, or magnetic resonance imaging (MRI), respectively. In particular, when therapeutics can be detected by various imaging tools, one imaging-capable therapeutic molecule can be used instead of two separate molecules of diagnostics and therapeutics. As imaging-capable therapeutics, fluorescent therapeutics, such as photosensitizers (e.g., pheophorbide a) and fluorescent drugs (e.g., doxorubicin), are well established.

### Mitochondria-targeting drug delivery

Active ingredients can target various subcellular organelles depending on their modes of action and their physico-chemical properties. For example, in general, cisplatin and pDNA are delivered into the nucleus to alkylate DNA and express mRNA, respectively. For paclitaxel and siRNA, they need to reach microtubules and target mRNA in the cytosol, respectively. Moreover, some drugs are required to be accumulated in lysosomes, mitochondria, and endoplasmic reticulum (ER). Among these subcellular organelles, this review focuses on mitochondria-targeting theranostics because mitochondria modulate significant physiological functions. In particular, mitochondria control the homeostasis of intracellular Ca^2+^ levels and oxidative stress and rule cell viability/death and signaling through bioenergy production, cellular differentiation/growth, the cell cycle, and cell necrosis/apoptosis/autophagy [4, 5]. Thus, their malfunction and dysfunction frequently cause unwanted growth or death of cells and lead to many neurodegenerative, neuromuscular, cardiac, and metabolic diseases and cancers [5, 6].

Despite the significance of delivering therapeutic molecules into mitochondria, as well as monitoring mitochondrial functions and morphology and therapeutic molecules localized in the mitochondria, the number of research articles on mitochondria-specific delivery was approximately 200 by 2014 [7]. Janus Green B was reported as the first mitochondria-staining dye in 1899 [8]. Moreover, the mitochondrial targeting and accumulating characteristics of the well-studied mitochondria-targeting triphenylphosphonium (TPP) and dequalinium (DQA) as molecules were first reported in 1969 [9] and 1987 [10], respectively. However, the first mitochondria-targeting drug conjugates or complexes using TPP and DQA were reported in 1995 [11] and 1998 [12], respectively. Studies on mitochondria-targeting drugs/diagnostics, drug/diagnostic conjugates, and drug/diagnostic delivery systems have been silently conducted by a few researchers by 2014. However, the interest in the topics has been substantially increasing since 2015 [7]. With the critical developments in biological and imaging analyses, chemical synthesis, and nanotechnology, various mitochondria-targeting moieties and their chemical conjugates and delivery systems have been extensively investigated. Nevertheless, the total number of research articles on mitochondria-specific delivery of therapeutics and imaging molecules remains less than 1000 [7]. Thus, mitochondrial drug delivery and particularly mitochondrial theranostics would be very hot research topics in the biomedical and pharmaceutical fields.

**Fig. 1:**
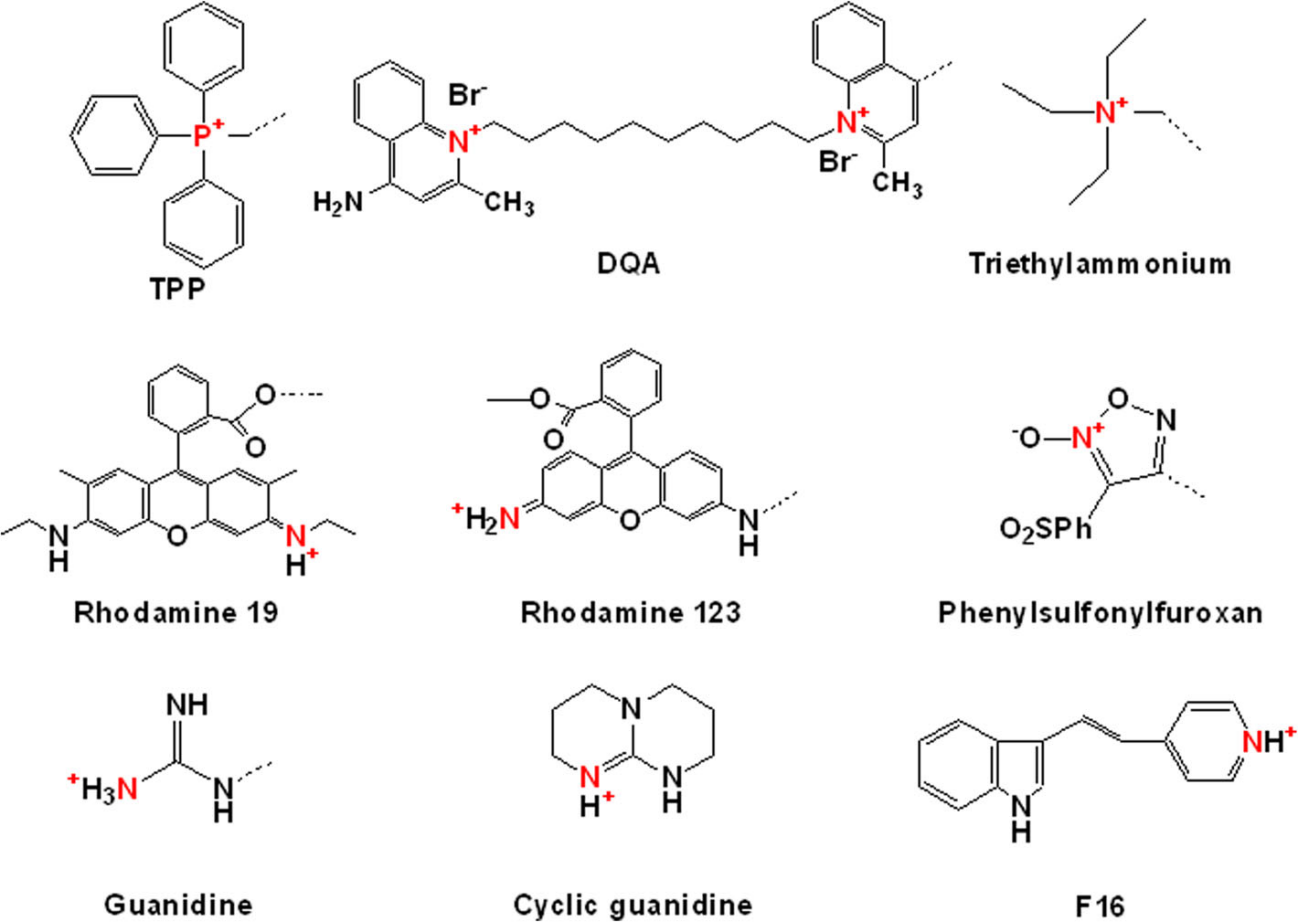
Examples of mitochondria-targeting chemicals

To date, many researchers have investigated various mitochondria-targeting moieties, including chemicals and peptides. As shown in Fig. 1, well-known mitochondria-targeting chemicals, such as TPP, DQA, (*E*)-4-(1*H*-Indol-3-ylvinyl)-*N*-methylpyridinium iodide (F16), rhodamine, and guanidine, have lipophilic cations or delocalized cations because these characteristics enable them to cross the mitochondrial membrane using the difference in the membrane potentials between the outer mitochondrial membrane and the inner mitochondrial membrane [7]. Furthermore, when introducing mitochondrial targetability into theranostics, it is possible to design three functionalities (i.e., treatment, imaging, and mitochondria-targeting abilities) in one conjugate and delivery system. Thus, this review will provide an overview of recent mitochondrial theranostic chemicals, chemical conjugates, and delivery systems.

### Mitochondria-targeting theranostics

**Fig. 2:**
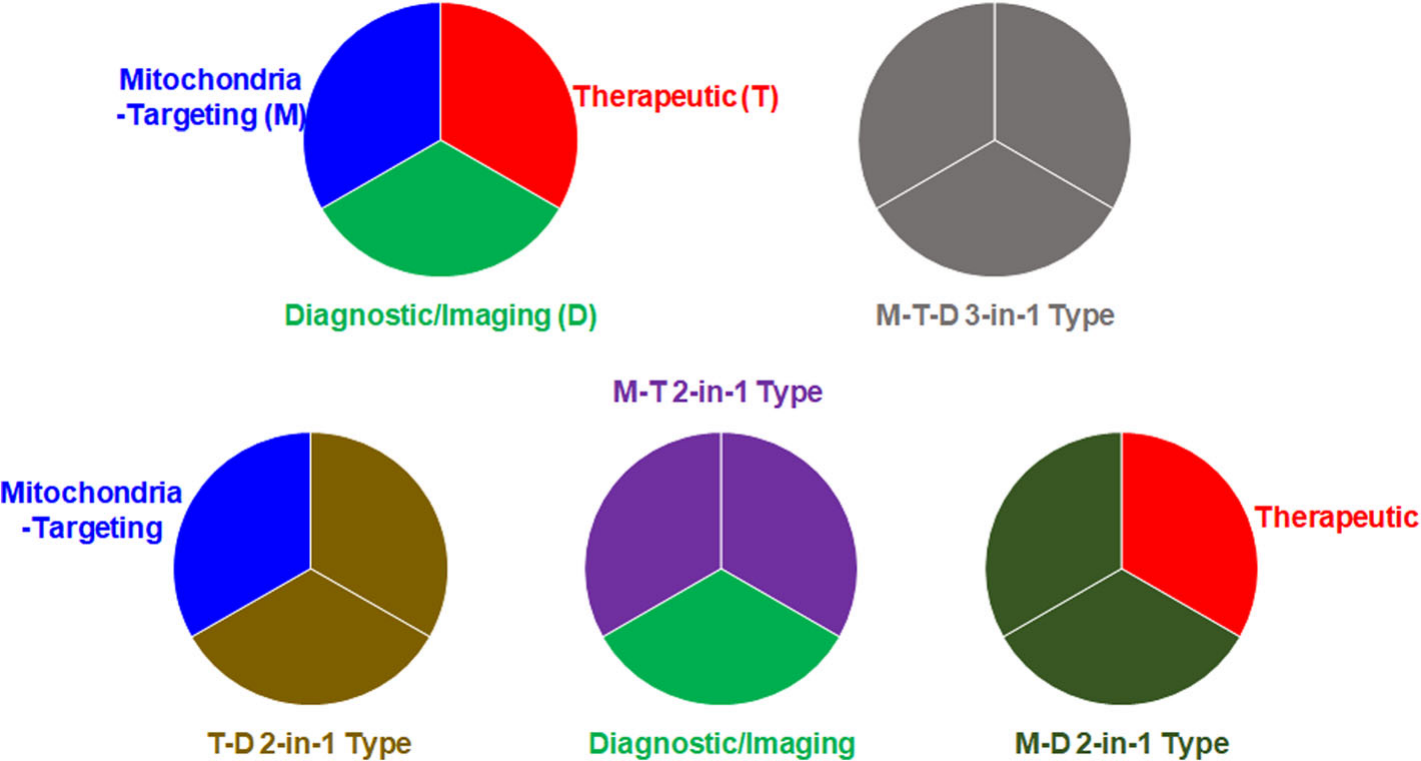
Types of mitochondria-targeting theranostics (Mito-Theranostics)

**Fig. 3:**
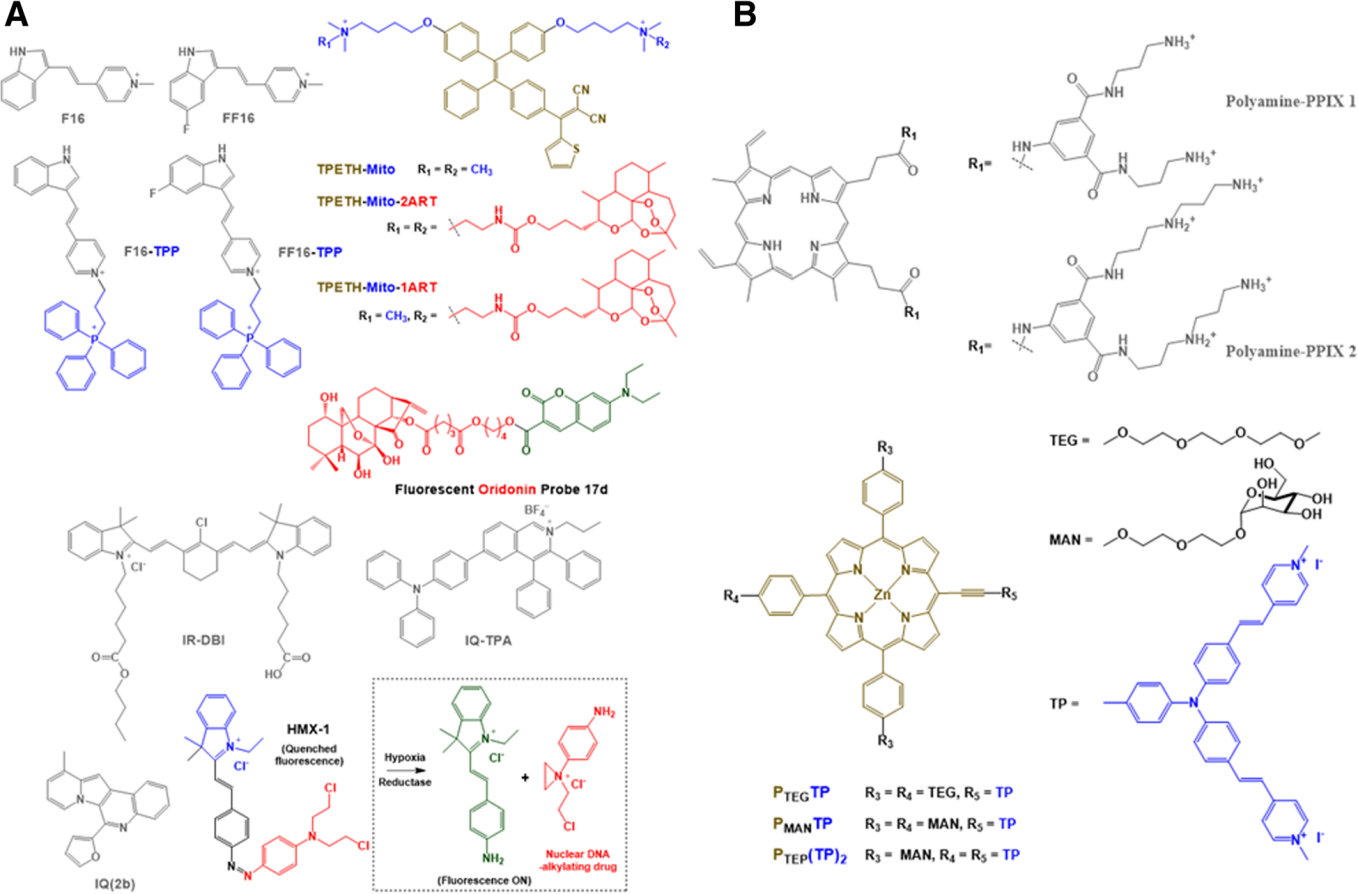
Chemical structures of Mito-Theranostic chemicals and chemical conjugates: (**a**) F16, FF16, F16-TPP, FF16-TPP, IQ(2b), IQ-TPA, IR-DBI, TPETH-Mito, TPETH-Mito-1ART, TPETH-Mito-2ART, fluorescent oridonin probe 17d, and HMX-1 and (**b**) polyamine-PPIX1, polyamine-PPIX2, P_TEG_-TP, P_MAN_TP, and P_TEG_(TP)_2_

As shown in Fig. 2, mitochondria-targeting theranostics have been designed by combining three different functional components, such as mitochondria-targeting components (M), therapeutic components (T), and imaging/diagnostic components (D) and have been prepared by mixing or conjugating single functional components with dual or triple functional components [13–15]. These components include chemical molecules, macromolecular structures, or nanosized systems. Thus, if each component has mono-functionality, three different components should be included in one mitochondria-targeting theranostic chemical conjugate or delivery system. However, if one component has dual functionalities (i.e., 2-in-1 typed components), such as M-T, M-D, or T-D, an additional D, T, or M functional component is chemically or physically introduced into the designed subcellular organelle-targeting theranostic conjugates/systems, respectively. If one component has all three functionalities of M, T, and D (i.e., 3-in-1 typed component), a single component intrinsically reaches the mitochondria, exerts a therapeutic effect and is simultaneously detected by imaging tools. In addition, to strengthen or synergize one functionality, two different components with the same functionality can be applied. Theoretically, tons of mitochondria-targeting theranostic chemical conjugates can be designed and synthesized by tons of combinations with three different components. Nevertheless, the reported numbers of mitochondrial-targeting theranostic chemical conjugates or nanostructures have not reached expectations. Limited examples are listed in Tables 1 and 2, and their chemical structures are shown in Fig. 3.

**Table 1:** Examples of mitochondria-targeting theranostic chemicals and chemical conjugates

Code Name	Mitochondria-targeting moieties (M)	Therapeutic agents (T)	Diagnostic/Imaging agents (D)	Therapeutic effects	Diagnostic/Imaging modes	Remarks	References
F16	F16	F16	F16	Apoptosis/necrosis-mediated tumor cell killing	Fluorescence Imaging	M-T-D 3-in-1 typed chemicals	[16]
F16 FF16 F16-TPP FF16-TPP	F16 FF16 F16, TPP FF16, TPP	F16 FF16 F16 FF16	F16 FF16 F16 FF16	Apoptosis/necrosis-mediated tumor cell killing	Fluorescence Imaging	M-T-D 3-in-1 typed chemicals or chemical conjugates Dual M type	[13]
F16–FU F16–OOC–FU F16–NHOC–FU F16–SS–FU	F16	F16, 5FU	F16	Apoptosis/necrosis-mediated tumor cell killing	Fluorescence Imaging	M-T-D 3-in-1 typed chemicals Dual T type Linkage effect	[17]
F16-BODIPY	F16	F16	F16, BODIPY	Apoptosis/necrosis-mediated tumor cell killing	Fluorescence Imaging	M-T-D 3-in-1 typed chemicals Dual D type	[18]
IQs	IQs	IQs	IQs	PDT	Fluorescence imaging	M-T-D 3-in-1 typed chemicals	[28]
IR-DBI	IR-DBI	IR-DBI	IR-DBI	PDT & PTT	NIR fluorescence imaging	M-T-D 3-in-1 typed chemicals	[29]
IQ-TPA	IQ-TPA	IQ-TPA	IQ-TPA	PDT	Fluorescence imaging	M-T-D 3-in-1 typed chemicals	[30]
Polyamine-Protoporphyrin IX (PPIX) conjugates	Polyamine-PPIX conjugates	PPIX	PPIX	PDT	Fluorescence imaging	M-T-D 3-in-1 typed chemical conjugates	[31]
Cationic porphyrin-triphenylamine (TP) hybrids	TP	porphyrin	porphyrin	PDT	Fluorescence imaging	T-D 2-in-1 typed chemical conjugates	[32]
TPETH-Mito TPETH-Mito-1ART TPETH-Mito-2ART	Mito (quaternary ammonium salts)	Tetraphenylethenethiophene (TPETH), Artemisinin (ART)	TPETH	Apoptosis, PDT	Fluorescence imaging	T-D 2-in-1 typed chemical conjugates	[33]
MitDt Compounds	TPP in MitDt	Indole group in MitDt	Indole group in MitDt	PDT	NIR fluorescence imaging	M-T-D 3-in-1 typed chemical conjugates	[34]
Fluorescent oridonin probe 17d	*N*,*N*-dialkyl-7-aminocoumarin derivative	oridonin	*N*,*N*-dialkyl-7-aminocoumarin derivative	Cell cycle arrest, apoptosis, and autophagy-mediated antitumor activity	Fluorescence imaging	M-D 2-in-1 typed chemical conjugates	[15]
HMX-1	Indole derivative in HMX-1	Aniline nitrogen mustard released from HMX-1	Fluorophore in HMX-1	Nuclear DNA-alkylation-mediated cell death	Fluorescence imaging	M-T-D 3-in-1 typed Pro-Theranostics	[35]

**Table 2:** Examples of mitochondria-targeting theranostic nanostructures

Code Name	Mitochondria-targeting moieties (M)	Therapeutic agents (T)	Diagnostic/Imaging agents (D)	Therapeutic effects	Diagnostic/Imaging modes	Remarks	References
Drug-loadable TPP-C NPs	TPP	Doxorubicin (model drug)	coumarin	Antitumor activity	Fluorescence imaging	Self-assembled NPs of chemical conjugates	[36]
PEG-(PhA)_2_ (PPA) NPs	Pheophorbide a (PhA)	PhA	PhA	ROS-mediated tumor cell killing	Fluorescence Imaging	M-T-D 3-in-1 typed NPs, Self-assembled NPs of chemical conjugates	[37]
PPA_n_-TPCL_4-n_ mixed NPs of PEG-(PhA)_2_ (PPA) and TPP-*b*-PCL-*b*-TPP (TPCL)	PhA, TPP	PhA	PhA	ROS-mediated tumor cell killing	Fluorescence Imaging	M-T-D 3-in-1 typed NPs, Self-assembled NPs of chemical conjugates, Dual M type	[37]
TICT	TPP	IR780, Ce6	IR780, Ce6	Photodynamic therapy (PDT) & Photothermal therapy (PTT)	Near-infrared (NIR) fluorescence imaging, photoacoustic imaging	T-D 2-in-1 typed NPs	[38]
IR-780-PEG-*b*-polyMTOS NPs	A methacrylic derivative of α-tocopheryl succinate (MTOS)	MTOS, IR-780	IR-780	MTOS-mediated apoptosis, IR-780-mediated PDT & PTT	NIR fluorescence imaging	M-T 2-in-1 & T-D 2-in-1 typed NPs	[39]
TAT-Ppa-UNCPs	TAT peptide (YGRKKRRQRRR)	Pyropheophorbide a (Ppa)	Ppa	ROS-mediated tumor cell killing	NIR fluorescence imaging	T-D 2-in-1 typed NPs	[40]
CDs-RB	Carbon dots (CD) composed of chitosan, ethylenediamine, and mercaptosuccinic acid	Rose Bengal (RB)	CD	ROS-mediated tumor cell killing	Fluorescence Imaging	M-D 2-in-1 typed NPs	[41]
Cdot-TPP-SNO	TPP	Cdot	Nitric oxide (NO)	NO-induced apoptosis	Fluorescence Imaging	NPs	[42]
ICG-loaded MMCNs	TPP	Indocyanine green (ICG)	ICG, Fe_3_O_4_	ICG-mediated PDT & PTT	NIR fluorescence imaging, Magnetic resonance (MR) imaging	Bimodal imaging NPs	[43]
C3N4-Fe-TPP NF/MB	TPP	Methylene blue (MB)	Fe^III^, MB	PDT	Fluorescence imaging, MR imaging	NSs	[44]

UNCPs (NaYbF_4_:Nd@NaGdF_4_:Yb/Er@NaGdF_4_ core-shell-shell upconversion nanoparticles); MMCNs (mitochondria-targeting composite nanoparticles composed of Fe_3_O_4_ core, poly(dopamine) (PDA) shell, ICG loaded mesoporous silica (mSiO_2_) and surface modified triphenylphosphonium (TPP); TICT (IR780/Ce6-loaded theranosome (TNS) self-assembled from DSPE-PEG-TPP); DSPE (1,2-distearoyl-sn-glycero-3-phosphoethanolamine); C_3_N_4_-Fe-TPP NF/MB (MB-loaded, Fe^III^-doped C_3_N_4_ nanofusiform (NF) with mitochondrial targeting TPP;

### Mitochondria-targeting theranostic chemicals and chemical conjugates

As shown in Table 1, most mitochondria-targeting theranostic chemicals and chemical conjugates are triple functional components (i.e., M-T-D 3-in-1 typed components) or dual functional components (e.g., M-D or T-D 2-in-1 typed components) with a mono-functional component. F16 is a representative of an M-T-D 3-in-1 typed component. In some cases, a few photosensitizer derivatives with lipophilic cations would be an M-T-D 3-in-1 typed component. In general, a delocalized cation (i.e., lipophilic cation) has been intentionally introduced into intrinsic theranostic photosensitizers.

**Fig. 4:**
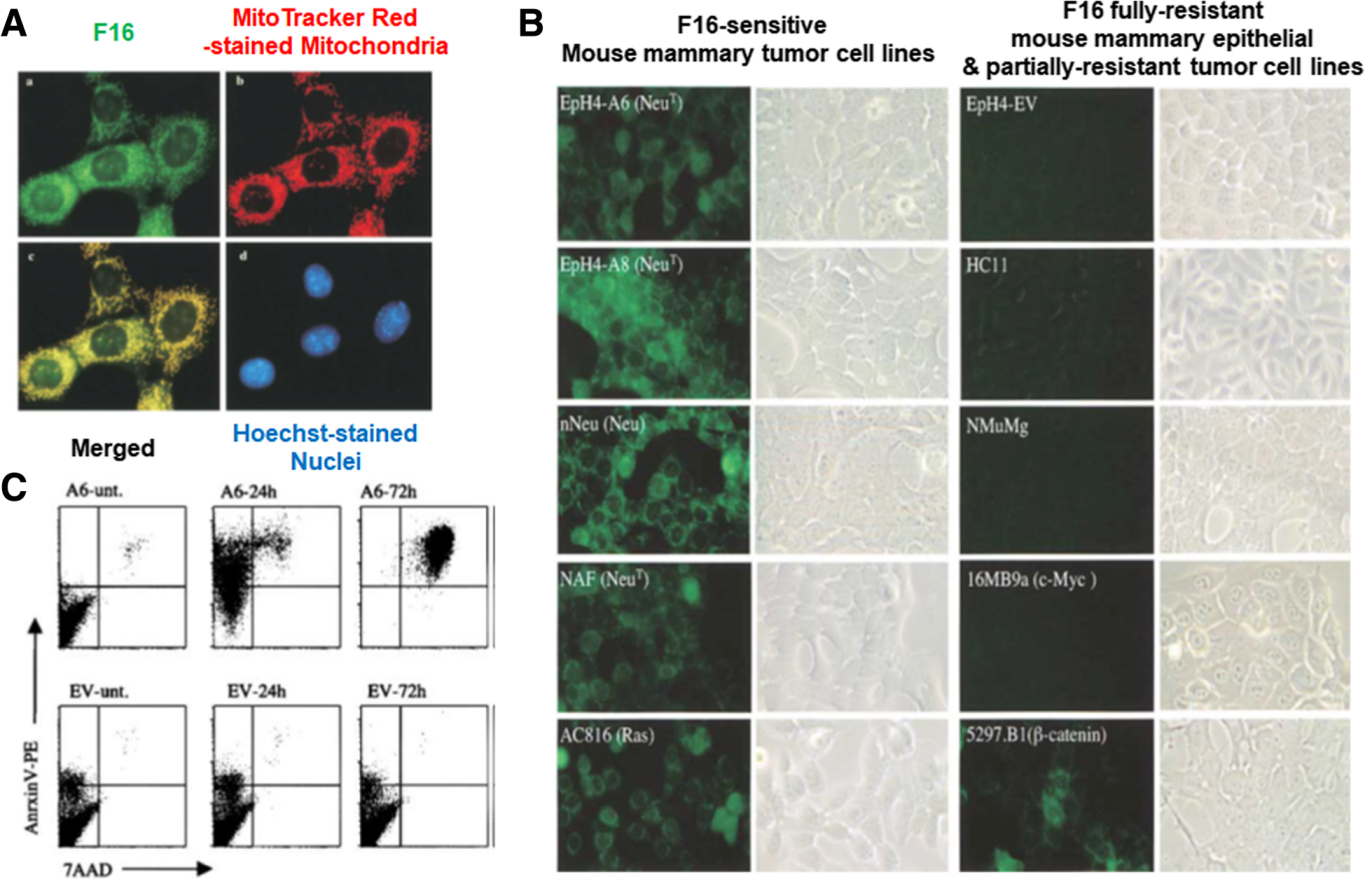
M-T-D 3-in-1 typed F16: (**a**) F16-mediated mitochondria-targeting activity in EpH4-A6 cells, (**b**) Fluorescence imaging of F16 in living cells, and (**c**) F16-mediated apoptosis activity in EpH4-A6 and control EV cells. Reproduced with permission from reference [16]; Copyright © 2002 Cell Press

For M-T-D 3-in-1 typed F16 mitochondria-targeting theranostic chemicals, Fantin et al. investigated the triple functionalities in 2002. The lipophilic cation and the indole linked pyridinium in the chemical structure (Figs. 1 and 3a) enabled its mitochondria-targeting ability and its intrinsic fluorescence, respectively, and F16 also showed killing activities against various cancer cells [16]. As shown in Fig. 4(a), when F16 was administered to EpH4-A6 cells (i.e., oncogene *neu*-overexpressing clone of EpH4 cell lines), the fluorescence of F16 was overlapped with the fluorescence of MitoTracker™ Red-stained mitochondria, which indicates the mitochondria-targeting activity of F16. However, the M-T-D functions of F16 were, in some cases, fully or partially activated or inactivated depending on the cells. After 24 h of treatment of F16 to various cell lines, its fluorescence imaging indicated that F16-sensitive cell lines (e.g., the EpH4 *neu*-overexpressing clones A6 and A8 and the v-Ha-*ras*-, *neu*-, and β-*catenin*-initiated tumor cell lines) had strong green fluorescence by F16 uptake (Fig. 4(b)). However, interestingly, F16 was not taken up or retained in immortalized, nontransformed mouse mammary epithelial cell lines (e.g., HC11, NMuMG, and EpH4-EV cells), human mammary epithelial MCF10A, and *c-myc* oncogene-initiated mouse tumor cell lines (Fig. 4(b)). These different F16 uptake activities influenced the cell proliferation activity. As shown in Fig. 4(c), F16-treated EpH4-EV cells did not show apoptotic and necrotic cell death; however, EpH4-A6 cells exhibited strong F16-mediated apoptosis. The evaluation of F16-mediated anti-proliferating activity using mouse tumor cells derived from oncogenes (e.g., *neu*-, v-Ha-*ras*-, β-*catenin*, or *c-myc*) and several human breast cancer cells showed that the growth inhibition of many oncogene-initiating tumor cells was strongly affected by F16: *neu*-oncogene expressing mouse breast cancer cells (e.g., NF980, SMF, NAF, n-Neu, Neu4145, NF324-2A, and NF324-1B), v-Ha-*ras*-oncogene expressing mouse breast cancer cells (e.g., AC816, AC711, AC236, and SH1.1), and human breast cancer cells (e.g., SKBR-3, T47D, ZR75, BT474, MCF-7, MDA-MB-436, MDA-MB-453, and MDA-MB-468). However, the growth of some tumor cells was not influenced by F16: v-Ha-*ras*-oncogene expressing mouse cancer cells (e.g., AC/Balb12, AC/Balb14, AC/Balb6.6, AC/p53^−^ #16, and AC/p53^−^ #19 for fibrosarcoma, AC260 for jaw cancers, AC99 for neck cancers, AC222 and AC/p53^−^ 4782 for intestinal cancers, and AC/p53^−^ #1 for salivary cancers), *c-myc*-oncogene expressing mouse breast cancer cells (e.g., 16MB9a, Myc 83, M158, and 13MA1a), and human breast cancer cells (e.g., MDA-MB-231 and MDA-MB-435). Although the exact reasons for the cellular resistance against F16 remain unclear, additional mitochondria-targeting moieties (e.g., TPP [13]), therapeutic agents (e.g., 5-fluorouracil (5FU) [17]), or imaging molecules (e.g., boron–dipyrromethene (BODIPY) [18]) have been introduced into F16-containing chemical conjugates for their improved mitochondria-targeting ability, improved tumor killing effect, or improved imaging activity, respectively. For enhanced M-T-D functions, one hydrogen chemically linked carbon at position 5 in the indole part of F16 was replaced with one fluorine, which resulted in the formation of FF16 [13].

Photosensitizers and their derivatives are intrinsic theranostic agents. Interestingly, although pyropheophorbide a (PPhA) derivatives that have an octyl-to-dodecyl ether at low concentrations [19] and *N*-aspartyl chlorin e6 [20] were mainly accumulated in lysosomes and Foscan® (*meta*-tetra[hydroxyphenyl]chlorin) mainly targeted the ER and Golgi apparatus [21], pheophorbide a (PhA) [22–25], a PhA derivative (e.g., DH-I-180-3) [26], a poly(ethylene glycol)-PhA conjugate [27], high dosed octyl-to-dodecyl ether PPhAs [19], and propyl-to-heptyl ether PPhAs [19] were mainly localized in mitochondria. Thus, some photosensitizers can possess M-T-D functionalities. Really, 6-(furan-2-yl)- and 6-(thiophen-2-yl) indolizino[3,2-c]quinolones (IQs) [28], IR-DBI [29], IQ-TPA [30], and polyamine-Protoporphyrin IX (PPIX) conjugates [31] (Fig. 3(a), (b), and Table 1) were designed to have delocalized lipophilic cations for mitochondria-targeting, light-triggered emission for fluorescence imaging, and light-triggered generation of reactive oxygen species (ROSs) for cell killing effects. For the cases in which it remains unclear whether photosensitizers have mitochondria-targeting activity, delocalized cations have been introduced in chemicals or chemical conjugates. For example, the introduction of triphenylamine (TP) into cationic porphyrin-TP hybrids (e.g., P_MAN_TP, P_TEG_TP, and P_TEG_(TP)_2_) enabled accumulation in mitochondria to selectively image the organelles and trigger their dysfunctions for cell death [32]. In the case of tetraphenylethenethiophene (TPETH)-Mito (i.e., quaternary ammonium) and its drug conjugates having artemisinin (ART) (i.e., TPETH-Mito-1ART and TPETH-Mito-1ART), their tumor killing effects were synergistically improved using two different anti-tumor drugs, such as TPETH and ART, via photodynamic therapy and chemotherapy, respectively [33].

**Fig. 5:**
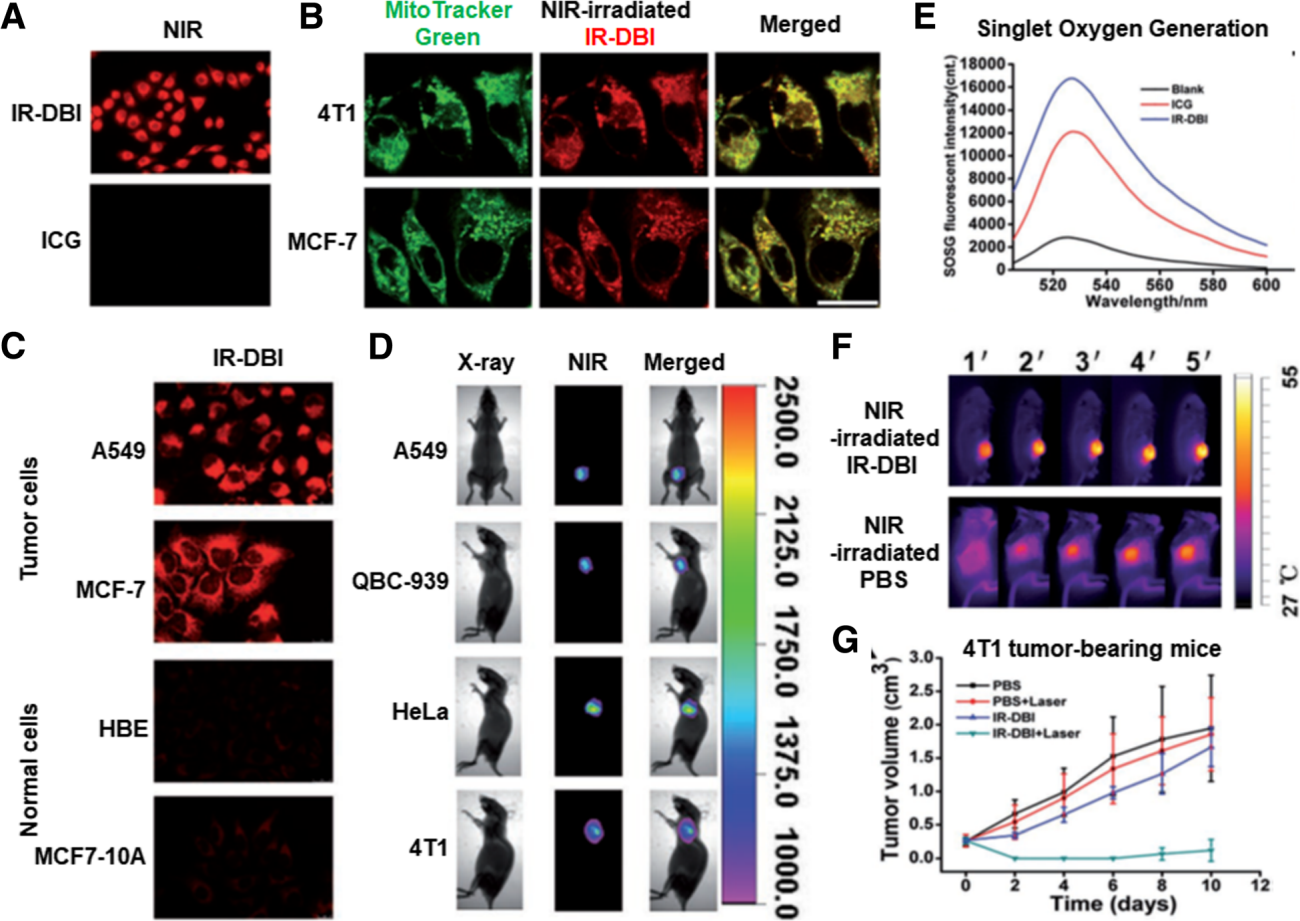
M-T-D 3-in-1 typed IR-DBI: (**a**) Fluorescence imaging of NIR-irradiated IR-DBI and control indocyanine green (ICG), (**b**) Mitochondria-targeting activity of IR-DBI in 4T1 and MCF-7 cell lines, (**c**) NIR fluorescence imaging of IR-DBI in tumor cell lines (A549 and MCF-7 cells) and normal cells (HBE and MCF-10A cells), (**d**) NIR fluorescence imaging of IR-DBI in tumor cell (A549, QBC-939, HeLa, and 4T1 cells)-bearing mice, (**e**) PDT activity (i.e., singlet oxygen generation) of NIR-irradiated IR-DBI and ICG, (**f**) PTT activity (i.e., temperature elevation) of NIR-irradiated IR-DBI in 4T1 tumor-bearing mice, and (**g**) Tumor inhibition of NIR-irradiated IR-DBI in 4T1 tumor-bearing mice. Reproduced with permission from reference [29]; Copyright © 2017 WILEY-VCH Verlag GmbH & Co. KGaA, Weinheim

Among the previously described mitochondria-targeting photosensitizers, Tan et al. designed IR-DBI as an alternative of theranostic indocyanine green (ICG) [29]. ICG is an FDA-approved NIR contrast agent with photodynamic therapy (PDT) and photothermal therapy (PTT). However, the poor cellular uptake and poor tumor-specific accumulation of ICG have limited its clinical applications. In contrast to the chemical structure of ICG, the chemical modification of a rigid cyclohexenyl group and the lipophilic cationic *N*-alkyl side chains in IR-DBI (Fig. 3(a)) enabled mitochondria-targeting and upgraded PDT and PTT, consequently leading to effective and selective tumor growth inhibition [29]. As shown in Fig. 5(a), when an NIR of 770 nm as an excitation wavelength was irradiated to IR-DBI or ICG-treated A549 cells, IR-DBI-treated A549 cells emitted substantially stronger fluorescence at 830 nm than ICG-treated A549 cells. In particular, in two breast cancer cell lines (i.e., 4T1 and MCF-7 cells), the intracellular localization of IR-DBI was strongly overlapped with the intracellular distribution of MitoTracker™ Green localized in the mitochondria, which indicates the strong mitochondria-targeting activity of IR-DBI (Fig. 5(b)). Interestingly, NIR fluorescence imaging of IR-DBI confirmed that IR-DBI accumulated selectively in tumor cells (e.g., A549 and MCF-7 cells); however, it was not detected in normal cells (e.g., HBE and MCF-10A cells) (Fig. 5(c)). The results suggest that IR-DBI specifically targets tumor cells and then further reaches their mitochondria and that IR-DBI could reduce the nonspecific cytotoxicity to normal cells. Beyond in vitro cell imaging, IR-DBI imaged solid tumors in A549, QBC-939, HeLa, or 4T1 subcutaneous tumor xenograft-bearing mice (Fig. 5(d)). Moreover, NIR-irradiated IR-DBI produced more singlet oxygen than control ICG (Fig. 5(e)) and heated body temperatures to approximately 54 °C in 4T1 tumor-bearing mice (Fig. 5(f)). As a result, PDT and PTT of NIR-irradiated IR-DBI almost completely inhibited tumor growth in 4T1 tumor-bearing mice (Fig. 5(g)). The research showed that M-T-D 3-in-1 typed IR-DBI as a small mitochondria-selective theranostic chemical could diagnose tumor cells and synergistically kill the cells with the multimodal therapeutic activities of chemotherapy, PDT, and PTT. In addition, Noh et al. designed a similar chemical structure of IR-DBI, but introduced TPP into indole containing photosensitizers, which ultimately led to the formation of MitDt compounds [34].

For the M-D 2-in-1 case, Xu et al. designed a fluorescent oridonin probe 17d [15]. The probe was synthesized by chemically linking an anticancer drug, oridonin, with an M-D 2-in-1 typed *N*,*N*-dialkyl-7-aminocoumarin derivative. A quaternary ammonium and a coumarin in the aminocoumarin derivative (Fig. 3(a)) endowed mitochondria-targeting activity and fluorescence imaging at an excitation/emission of 425 nm/454 nm, respectively. In particular, oridonin in the conjugate destabilized mitochondrial membrane potentials, triggered to release cytochrome c, and induced apoptotic cell death. As a result, the fluorescent oridonin probe 17d showed approximately 2.04-fold, 5.85-fold, and 8.9-fold higher killing activities than oridonin in A549, HepG2, and HeLa cells, respectively.

Interestingly, Hu et al. designed mitochondria-targeting pro-theranostic chemical conjugates [35]. In Fig. 3(a), the indole derivative in HMX-1 delivered nonfluorescent HMX-1 (i.e., a pro-theranostic) into mitochondria. A hypoxic condition triggered the break of an azo bond in an azobenzene spacer, which functions to quench a fluorescent probe and deactivate an aniline nitrogen mustard, and then released the fluorophore and the active drug for imaging mitochondria and killing cells, respectively.

### Mitochondria-targeting theranostic nanosystems

Using various nanostructures, such as micelles, vesicles (e.g., polymersomes and liposomes), nanogels, mesoporous nanospheres, nanosheets (NSs), and nanoparticles (NPs), mitochondria-targeting theranostic nanosystems can be constructed. Their major component materials are not limited, and polymers, silica, metals, lipids, carbon, or their hybrids have been used. Examples are listed in Table 2.

First, amphiphilic chemical conjugates, polymers, lipids, or polymer-lipid hybrids, such as a TPP-linked coumarin probe (TPP-C) composed of a hydrophilic TPP and a hydrophobic coumarin fluorophore [36], PEG-*b*-(PhA)_2_ (PPA) polymers composed of a hydrophilic PEG and two photosensitizing PhA molecules (i.e., M-T-D 3-in-1 typed theranostics), and a 3-arm linking bridge with single carbon-carbon bonds or disulfide bonds [37], TPP-PEG_2000_–1,2-distearoyl-*sn*-glycero-3-phosphoethanolamine (DSPE) polymer-lipid conjugates composed of hydrophilic mitochondria-targeting TPP-PEG_2000_ and hydrophobic DSPE [38], and carboxylated PEG-*b*-poly (mitochondria-targeting derivate of α-tocopheryl succinate (α-TOS)) (polyMTOS) composed of hydrophilic carboxylated PEG and hydrophobic mitochondria-targeting anticancer polyMTOS (i.e., M-T 2-in-1 typed theranostics) [39] have been used to construct nanosized micelles or vesicles in aqueous environments. Among these examples, PPA NPs do not require additional components because of their intrinsic M-T-D triple functionalities. However, M-D 2-in-1 typed TPP-C NPs and M-T 2-in-1 typed carboxylated PEG-*b*-polyMTOS NPs must chemically or physically load therapeutic components and diagnostic components, respectively. In the case of TPP-PEG_2000_-DSPE NPs, the two components of T and D are required.

**Fig. 6:**
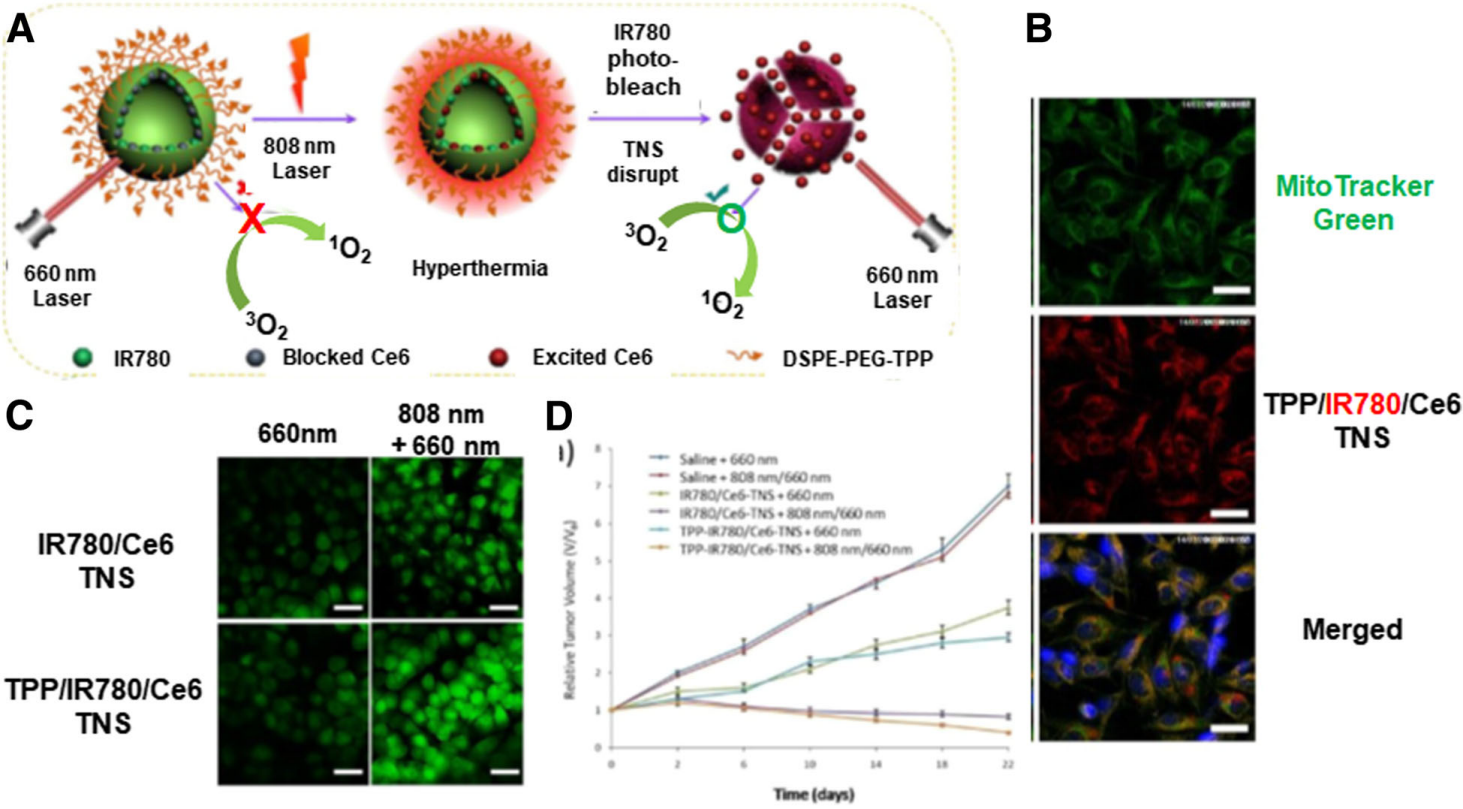
Dual-mode imaging guides multifunctional theranosomes (TNS): (**a**) Design concept of TPP/IR780/Ce6 TNS, (**b**) Mitochondria-targeting activity of TPP/IR780/Ce6 TNS in HeLa cells after 6 h of incubation, (**c**) Intracellular ROS generation of light-irradiated TPP/IR780/Ce6 TNS detected by 2′,7′-dichlorodihydrofluorescein diacetate (DCFH-DA) in HeLa cells, and (**d**) Tumor inhibition of laser-irradiated TPP/IR780/Ce6 TNS in HeLa tumor-bearing mice. Reproduced with permission from reference [38]; Copyright © 2018 American Chemical Society

Wang et al. designed a mitochondria-targeting, dual-mode imaging guided multifunctional theranosome (TNS) (Fig. 6) [38]. The vesicle-typed TNS was mainly constructed by a self-assembly of TPP-PEG_2000_-DSPE polymer-lipid conjugates, and mitochondria-targeting TPP was located on the surface of TNS due to the hydrophilicity of TPP-PEG_2000_. During the preparation of TNS, two photosensitizers (i.e., IR780 and Ce6) were physically encapsulated in the TNS, which resulted in the formation of TPP/IR780/Ce6 TNS (TICT). The TICT was designed to produce PTT and PDT for effective tumor killing and both NIR fluorescence and photoacoustic imaging. In particular, the sequence of laser irradiation is very significant. If a 660 nm laser for activating Ce6 is first irradiated to TICT, the blocked status of Ce6 in TICT cannot produce singlet oxygen. Thus, as shown in Fig. 6(a), 808 nm NIR light is first irradiated to TICT to produce heat and PTT activity and disrupt lysosomes. TICT escaped from the lysosomes targets mitochondria using TPP in TICT and is then disrupted under hyperthermia. The integrity loss of TICT releases Ce6, and a 660 nm laser is irradiated to the released Ce6 to generate singlet oxygen and activate PDT. The sequential activation of PTT and PDT by two different photosensitizers in TICT effectively kills tumor cells and monitors body temperatures, NIR fluorescence, and photoacoustic signals. In contrast to the poor mitochondrial localization of IR780/Ce6 TNS (ICT), the red fluorescence of IR780 in TICT was perfectly merged with the green fluorescence of MitoTracker™ Green-stained mitochondria (Fig. 6(b)). For singlet oxygen generation, when only 660 nm light was irradiated, TICT and ICT produced low ROS levels. However, when sequentially irradiating 808 nm and then 660 nm light, both TICT and ICT produced more intracellular ROS levels than the cases of only 660 nm irradiation. The singlet oxygen levels produced by TICT were substantially higher than those produced by ICT (Fig. 6(c)). Interestingly, the designed theranostic nanosystems generated heat to increase body temperature for effective PTT. When an 808 nm NIR laser was on for 2 min, IR780 generated heat, and TICT and ICT in a test tube made 59.4 °C and 58.2 °C of T_max_, respectively. The heat generation of TICT was substantially more effective than that of free IR780. Moreover, when giving the irradiation cycle through the switch “ON” and “OFF” of the 808 nm NIR laser, IR780 in TICT slowly lost the heat generation activity in contrast to the rapid loss of free IR780. NIR fluorescence imaging generated by IR780 in TICT monitored the biodistribution of TICT in tumor-bearing mice. Compared to free IR780/Ce6, TICT exhibited a stronger NIR fluorescence, and its fluorescence was maintained for a longer time. Interestingly, the photoacoustic imaging produced by TICT was continuously increased by 24 h, and its signal intensity was approximately 2.5-fold higher than that of free IR780/Ce6 at 24 h. Moreover, although TICT did not contain contrast agents, the photoacoustic imaging of TICT was better than its ultrasound imaging. Although mice were exposed to the 808 nm NIR laser for 5 min, the 808 nm-irradiated TICT produced substantially higher body temperatures in tumor sites than the 808-irradiated free IR780/Ce6. Combining PTT and PDT resulted in TICT-induced complete growth inhibition of HeLa tumors in mice (Fig. 6(d)). Thus, TICT has multiple potentials for mitochondria-targeting, synergistic tumor killing via the dual modes of PTT and PDT, as well as multiple tumor imaging by NIR fluorescence, photoacoustic signals, and temperatures.

**Fig. 7:**
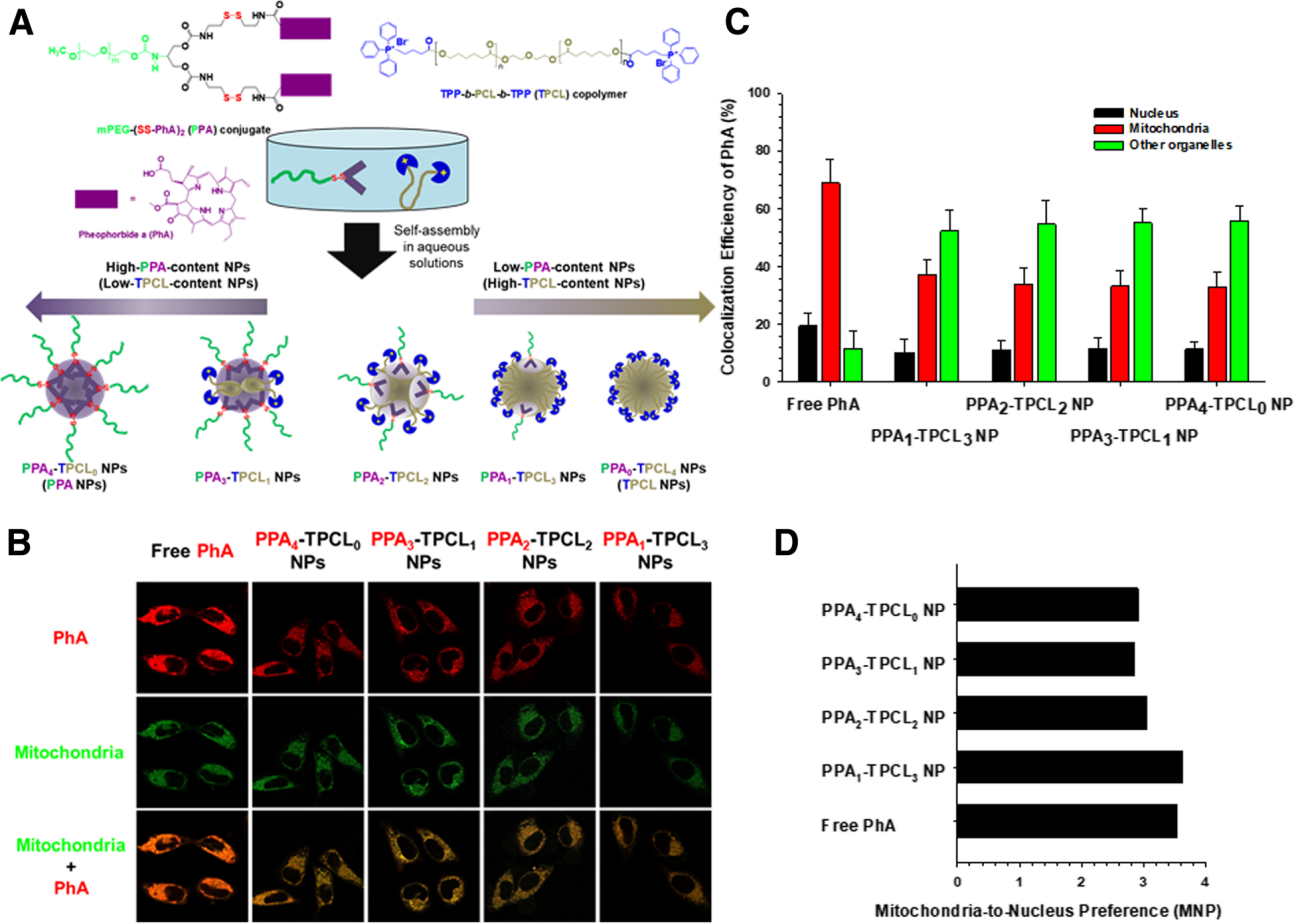
M-T-D 3-in-1 typed PPA_n_-TPCL_4-n_ nanoparticles (NPs): (**a**) preparation of PPA_n_-TPCL_4-n_ NPs, (**b**) Mitochondria-targeting activity and fluorescence imaging of PPA_n_-TPCL_4-n_ NPs in HeLa cells after 4 h of incubation, (**c**) Colocalization efficiency of PhA delivered by PPA_n_-TPCL_4-n_ NPs in HeLa cells after 4 h of incubation, and (**d**) Mitochondria-to-Nucleus preference (MNP) of PPA_n_-TPCL_4-n_ NPs in HeLa cells after 4 h of incubation. Reproduced with permission from reference [37]; Copyright © 2017 Elsevier B.V

In general, photosensitizers have two functionalities, including therapeutic effects and imaging activities. Their theranostic activities enable the design of multifunctional and multipotent organelle-targeting theranostics with their intrinsic subcellular localization. In particular, as previously discussed, some photosensitizers showed mitochondria-targeting activities. Choi et al. attempted to confirm whether one photosensitizer, PhA, can target specifically to mitochondria and evaluated whether the mitochondria-targeting activity of PhA is higher than that of the well-known mitochondria-targeting TPP [37]. To answer these two questions, two different amphiphilic polymers (i.e., TPP-*b*-poly(ε-caprolactone) (PCL)-*b*-TPP (TPCL) polymers and PPA polymers) were used to make their mixed micellar structures (i.e., PPA_n_-TPCL_4-n_ nanoparticles (NPs)) (Fig. 7(a)). The fluorescence of free PhA was perfectly overlapped with the green fluorescence of MitoTracker™ Green-stained mitochondria (Fig. 7(b)). Although the fluorescent intensities of PhA delivered by PPA_n_-TPCL_4-n_ NPs were lower than those of free PhA, the mitochondrial distribution of PPA_n_-TPCL_4-n_ NPs was confirmed by comparing with the intracellular distribution of MitoTracker™ Green (Fig. 7(b)). However, using confocal images, a colocalization analysis of PhA delivered by PPA_n_-TPCL_4-n_ NPs indicated that many PPA_n_-TPCL_4-n_ NPs remained entrapped in endolysosomal compartments due to their poor endosomal escaping activities (Fig. 7(c)). Nevertheless, the mitochondria-to-nucleus preferences (MNPs) of PPA_n_-TPCL_4-n_ NPs were approximately 3~ 3.5 and were similar or slightly lower than that of free PhA (Fig. 7(d)). The research showed that PhA can be used as a mitochondria-targeting theranostic and its mitochondria-targeting activity is almost equivalent to that of TPP.

TPP-C NPs were also formed by a self-assembly of TPP-coumarin chemical conjugates in aqueous solutions [36]. The formed mitochondria-targeting, fluorescent TPP-C NPs can load various hydrophobic therapeutic molecules. Interestingly, although approximately 80% of TPP-C NPs were localized in endolysosomes at 30 min of incubation, approximately 80% of TPP-C NPs escaped from the endolysosomes and then reached the mitochondria at 2 h of incubation. In the case of carboxylated PEG-*b*-polyMTOS, the polymer was chemically linked with an NIR photosensitizer, IR780 with PTT and PDT at the end carboxylic acid of the polymer [39]. A self-assembly of IR780-PEG-*b*-polyMTOS polymers in aqueous solutions formed phototherapeutic IR-NP. Furthermore, when IR780-PEG-*b*-polyMTOS polymers were self-assembled in aqueous solutions, additional IR780 molecules were added into the formed nanostructure, IR-NP-eIR. It is unclear whether the phototherapeutic NPs targeted to the mitochondria because the location of mitochondria-targeting polyMTOS in the NPs could be at their hydrophobic core. Nevertheless, IR-NP-eIR generated more heat by NIR irradiation than IR-NP and killed more tumor cells than IR-NP.

In addition, after constructing self-assembled nanosystems from amphiphilic materials, organelle-specific targeting moiety and components for therapeutics, diagnostics, or theranostics are introduced into the nanosystems to produce mitochondria-targeting theranostic NPs. Zhang et al. first made upconversion NPs (UCNPs) using amphiphilic poly(maleic anhydride-*alt*-1-octadecene) (C18PMH)-*b*-PEG-NH_2_, and the mitochondria-targeting TAT peptide was then chemically linked on the end functional amine of UCNPs [40]. The formed TAT-UCNPs physically encapsulated a theranostic PPhA derivative (i.e., Ppa) for PDT-mediated tumor killing and NIR fluorescence imaging.

**Fig. 8:**
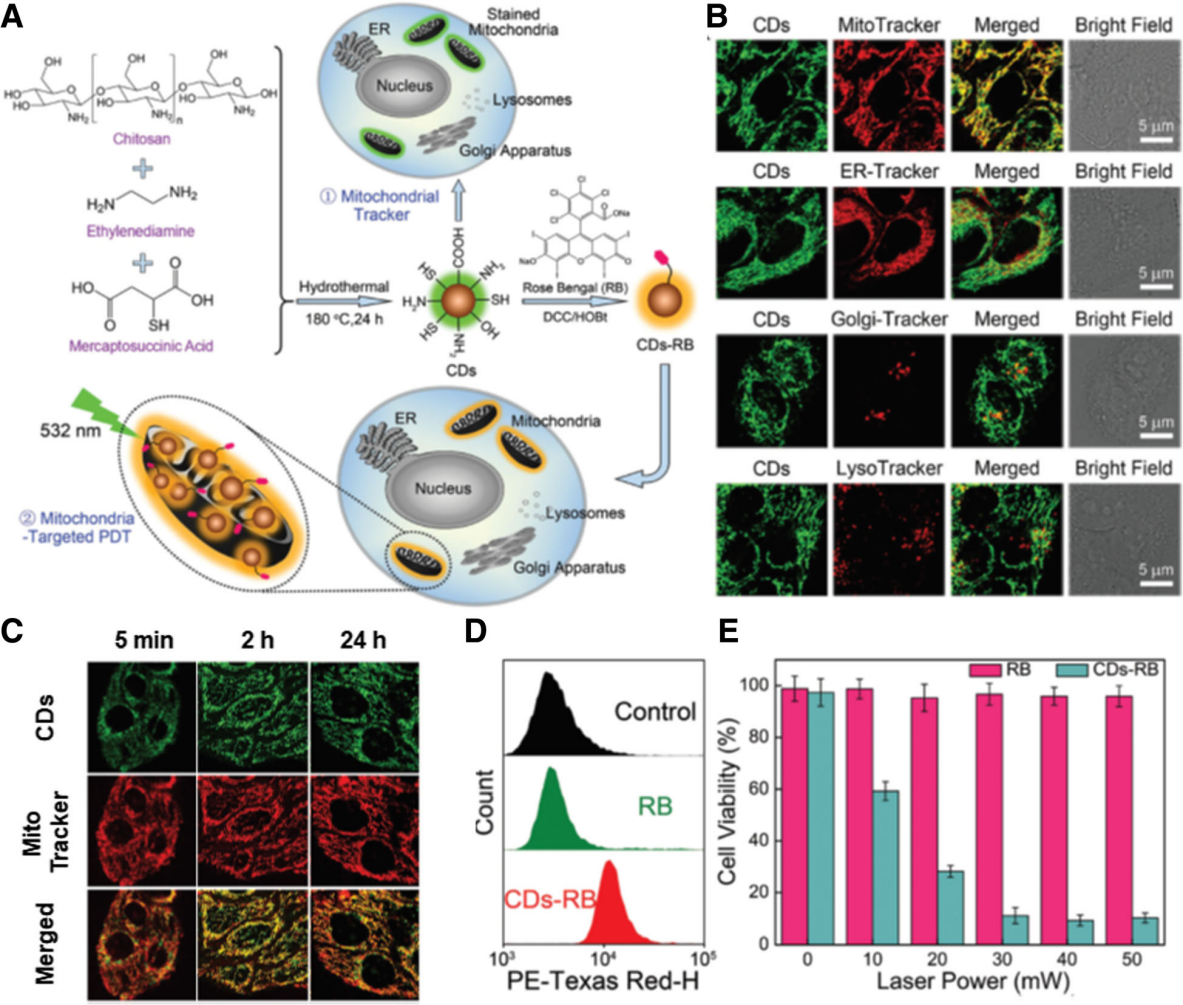
Rose bengal (RB)-loaded carbon quantum dots (CDs): (**a**) Synthetic route of CDs and design concepts of RB-loaded CDs (CDs-RB) in cells, (**b**) Subcellular localization of fluorescent CDs in MCF-7 cells after 30 min of incubation, (**c**) Mitochondria tracking activity of CDs in MCF-7 cells, (**d**) Cellular uptake of RB delivered by CDs-RB in MCF-7 cells, and (**e**) Cytotoxicity of light irradiated-CDs-RB in MCF-7 cells after an irradiating 532 nm laser for 5 min. Reproduced with permission from reference [41]; Copyright © 2017 Royal Society of Chemistry

Second, organic NPs can be prepared by in situ synthesis. For example, a one-step hydrothermal method enabled the three materials of chitosan, ethylenediamine, and mercaptosuccinic acid as carbon sources to form nanosized carbon quantum dots (CDs) [41]. The formed CDs possess intrinsic green fluorescence, which may be used for intracellular tracking, such as a mitochondrial tracker. In particular, when chemically conjugating a photosensitizer, rose bengal (RB) on CDs, the formed CDs-RB can be considered mitochondria-targeting theranostic nanosystems (Fig. 8(a)). Colocalization studies of CDs with various organelle-trackers (e.g., MitoTracker™, ER-Tracker™, Golgi-Tracker™, and LysoTracker™) showed that most CDs were accumulated in mitochondria; however, many CDs were also distributed in the ER. This less organelle-specificity could be caused by complicate functional groups, such as amine, hydroxyl, thiol, and carboxylic acid, on the surface of CDs. Nevertheless, the mitochondria tracking activity of CDs was activated at a very early incubation time (e.g., 5 min) and remained for 24 h (Fig. 8(c)). For mitochondria-targeting theranostic CDs-RB, intracellular RB delivery with CDs-RB was substantially more effective than free RB (Fig. 8(d)) and caused substantially more tumor killing activities than free RB (Fig. 8(e)). In addition, Xu et al. constructed CDs using citric acid and ethylenediamine (i.e., Cdot) [42]. The Cdot also showed intrinsic fluorescence. However, for mitochondria-targeting activity, TPP was chemically introduced on the end functional group of Cdot. Moreover, nitric oxide (NO)-releasing therapeutic molecules were chemically modified on the surface of Cdot. The resultant Cdot-TPP-SNO was selectively accumulated in mitochondria and then light-induced NO release from the nanosystems killed tumor cells.

Third, noncarbon-based nanostructures have also been applied for mitochondria-targeting theranostic systems. Guo et al. designed mitochondria-targeting composite NPs (MMCNs) composed of a spherical magnetite (Fe_3_O_4_) core, polydopamine (PDA) inner shell, mesoporous silica (mSiO_2_) outer shell, and surface-decorated cell-targeting transferrin (Tf) and mitochondria-targeting TPP [43]. The lack of therapeutic molecules was solved by introducing a photosensitizer, ICG. Under NIR irradiation, ICG-loaded MMCNs generated more heat and cell killing activities than Fe_3_O_4_@PDA@mSiO_2_ and Fe_3_O_4_@PDA. In particular, ICG-loaded MMCNs imaged tumors by ICG-mediated NIR fluorescence, Fe_3_O_4_-mediated T_2_-MRI, and ICG/Fe_3_O_4_-mediated photothermal signals. In addition, Ma et al. constructed TPP-mediated mitochondria-targeting Fe^3+^-doped two dimensional (2D) C_3_N_4_ nanofusiform (NF) [44]. After forming 2D graphitic phase C_3_N_4_ NSs, Fe^3+^ was doped on the NSs. The mitochondria-targeting TPP was then chemically introduced into Fe^3+^-doped C_3_N_4_ NF, and a photosensitizing methylene blue (MB) was also physically loaded into the NF. The resultant C_3_N_4_-Fe-TPP NF/MB showed substantially more mitochondria-targeting activities and light-irradiated higher cell-killing activities, as well as more tumor growth inhibition than C_3_N_4_-Fe NF/MB and C_3_N_4_ NS/MB. Furthermore, the doped Fe^3+^ on C_3_N_4_-Fe-TPP NF/MB enabled imaging of tumor areas in mice by T_1_-weighted MRI.

## Conclusions and future perspectives

In summary, the current research on mitochondria-targeting theranostic chemicals, chemical conjugates, or nanosystems is substantially increasing with the significance of subcellular organelle specificity and simultaneous modes of therapy and diagnosis. In the case of mitochondria-targeting theranostic chemicals and chemical conjugates, mitochondria-targeting lipophilic cations have been frequently introduced into the chemicals and chemical conjugates, resulting in the synthesis of M-T-D 3-in-1 typed materials. These reasons caused many photosensitizers to be applied to design mitochondria-targeting theranostics, and the use of well-known mitochondria-targeting moieties (Fig. 1) has been limited. In particular, although chemical synthesis using three different M, T, and D components enables various combinations and many new mitochondria-targeting theranostic chemical conjugates, the combination or design-based chemical syntheses have been limited due to the complexity, difficulty, and inconvenience of their syntheses. In addition, although lipophilic cations of mitochondria-targeting moieties could distinguish differences in the mitochondrial membrane potentials between tumor cells and normal cells, in some cases, the resistance of the designed chemicals (e.g., F16) could reduce their applications.

As alternatives of chemicals or chemical conjugates, mitochondria-targeting nanosystems composed of various materials have been considered. The nanosystems could detour the resistance mechanisms of chemicals. Physical loading of therapeutic molecules and diagnostic molecules in nanosystems could extend various combinations of T and D in mitochondria-targeting nanosystems. These platform-based nanosystems could minimize the complexity, difficulty, and inconvenience of chemical synthesis. Nevertheless, the current studies have often employed TPP as a mitochondria-targeting moiety and photosensitizers due to their intrinsic theranostic characteristics. Although TPP has good mitochondria-targeting activity, its positive charge could limit its use because many negatively charged blood components could cause TPP-based theranostic nanosystems to be useless via the formation of nonspecific aggregates. Thus, alternatives to TPP, such as negatively charged or hydrophilic, neutral charged mitochondria-targeting moieties, should be investigated. Furthermore, the lack of an endosomal escaping ability of the nanosystems could reduce their mitochondria-targeting effects because nanosystems, in general, follow endocytic pathways for their cellular entry. Thus, to produce effective mitochondria-targeting nanosystems, the nanosystems should equip endosomolytic functions. In addition, although NIR photosensitizers enable imaging of NIR fluorescence, photoacoustic signals, and photothermal signals, their corresponding diagnostic materials should be considered for other imaging tools, such as MRI, PET, and CT.
